# Effect of circular RNA, mmu_circ_0000296, on neuronal apoptosis in chronic cerebral ischaemia via the miR-194-5p/Runx3/Sirt1 axis

**DOI:** 10.1038/s41420-021-00507-y

**Published:** 2021-05-29

**Authors:** Keyu Huang, Chunqing Yang, Jian Zheng, Xiaobai Liu, Jie Liu, Dongfang Che, Yixue Xue, Ping An, Di Wang, Xuelei Ruan, Bo Yu

**Affiliations:** 1 Department of Neurosurgery, Shengjing Hospital of China Medical University, Shenyang, China; 2 Key Laboratory of Neuro-oncology in Liaoning Province, Shenyang, China; 3 Liaoning Clinical Medical Research Center in Nervous System Disease, Shenyang, China; 4 Department of Neurobiology, School of Life Sciences, China Medical University, Shenyang, China; 5 Key Laboratory of Cell Biology, Ministry of Public Health of China, China Medical University, Shenyang, China; 6 Key Laboratory of Medical Cell Biology, Ministry of Education of China, China Medical University, Shenyang, China

**Keywords:** Apoptosis, Stroke, Molecular neuroscience

## Abstract

Chronic cerebral ischaemia (CCI) is a common pathological disorder, which is associated with various diseases, such as cerebral arteriosclerosis and vascular dementia, resulting in neurological dysfunction. As a type of non-coding RNA, circular RNA is involved in regulating the occurrence and development of diseases, such as ischaemic brain injury. Here, we found that HT22 cells and hippocampus treated with CCI had low expression of circ_0000296, Runx3, Sirt1, but high expression of miR-194-5p. Overexpression of circ_0000296, Runx3, Sirt1, and silenced miR-194-5p significantly inhibited neuronal apoptosis induced by CCI. This study demonstrated that circ_0000296 specifically bound to miR-194-5p; miR-194-5p bound to the 3′UTR region of Runx3 mRNA; Runx3 directly bound to the promoter region of Sirt1, enhancing its transcriptional activity. Overexpression of circ_0000296 by miR-194-5p reduced the negative regulatory effect of miR-194-5p on Runx3, promoted the transcriptional effect of Runx3 on Sirt1, and inhibited neuronal apoptosis induced by CCI. mmu_circ_0000296 plays an important role in regulating neuronal apoptosis induced by CCI through miR-194-5p/Runx3/Sirt1 pathway.

## Introduction

Chronic cerebral ischaemia (CCI) is a progressive neurodegenerative process caused by long-term insufficient cerebral blood perfusion. It is one of the main risk factors of some neurodegenerative diseases, such as vascular dementia (VD) and Alzheimer’s (AD)^1,2^. Hippocampus is one of the most important nerve centres for learning and memory and is closely related to advanced functions, such as learning, memory, and cognition. Neurons in the hippocampal CA1 area are extremely vulnerable to ischaemia or hypoxia^3,4^. As a result, studying the mechanism of hippocampal neuron damage caused by CCI has important theoretical significance and application value.

There are many models for studying CCI, among which the 2-VO model is widely used^5,6^. However, in the 2-VO model, cerebral blood flow decreases sharply in the early stage of ischaemia and is gradually recovered in the middle and late stages. This process is inconsistent with the hemodynamic changes in patients with CCI^7^. Therefore, in this study, we used a new model of CCI: ameroid constrictor (AC) model. AC consists of an outer titanium ring and inner casein. When the common carotid artery is nested in AC, the casein absorbs water and expands, gradually narrowing the blood vessels, resulting in a slow and steady decline in cerebral blood flow^8^. Compared with 2-VO and other models, the AC model simulates the changes in clinical haemodynamics of patients with CCI better^9^.

Circular RNAs (circRNAs) are a class of single-stranded, closed-loop, endogenous non-coding RNAs (ncRNAs), without 5′end caps and 3′end poly-A tails, with high stability. The size varies from 100 b to 4 kb^10^. Studies have shown that circRNAs have many important functions. For example, a circRNA can serve as a molecular sponge for miRNA, regulate the expression of target genes, and participate in regulating the proliferation, migration, invasion, and apoptosis of various cells^11–14^. circNCX1 binds to miR-133a-3p, weakens the regulation of its target gene CDIP1, downregulates its expression, and promotes cardiomyocyte apoptosis caused by oxidative stress^15^. circ_0109320 inhibits the expression of miR-395, thereby upregulating its target gene E2F7, regulating the proliferation, migration, invasion and apoptosis of lung tumour cells^16^. circDLGAP acts as a molecular sponge for miR-143, increases the expression of HECTD1, improves neurological damage induced by ischaemic stroke, and reduces the area of cerebral infarction^17^. circ_0000296 (mmu_circ_0000296, circ-Nufip2), located on chromosome 11 and having a total length of 1713bp, is derived from the cyclisation of linear RNA nuclear fragile X mental retardation protein-interacting protein 2 (Nufip2).

MicroRNAs (miRNAs) are composed of about 20 nucleotides. They are highly conserved small ncRNAs that regulate not only normal physiological functions, such as brain neurogenesis and synapse formation^18,19^ but also neurodegeneration. Ischaemia and hypoxia play an important role in pathophysiological processes^20,21^. miR-194 is involved in the morphogenesis of inner ear spiral ganglion neurons by targeting RhoB recombinant actin cytoskeleton^22^. miR-378 can reduce intestinal mucosal cell apoptosis by inhibiting caspase-3 activation^23^. miR-93 reduces ischaemic nerve injury by activating the Nrf2/HO-1 antioxidant pathway^24^. The application of the bioinformatics software starBase revealed that circ_0000296 and miR-194-5p have potential binding sites, but the expression and function of circ_0000296 and miR-194-5p in CCI have not been reported.

Runt-related transcription factor 3 (Runx3) is a regulatory factor that plays a vital role in cell development by participating in a series of important physiological and pathological processes of organisms, such as cell growth, proliferation, migration, apoptosis, and angiogenesis^25,26^. Runx3 inhibits the proliferation, migration, and invasion of gastric cancer cells through the miR-182/HOXA9 pathway^27^. It inhibits glioma cell proliferation and invasion and induces cell cycle arrest through the β-catenin/TCF-4 signalling pathway^28^. Runx3 also regulates hypoxia-induced endothelial-to-mesenchymal transition of human cardiac microvascular endothelial cells (CMECs)^29,30^. The starBase predicted that there was a potential binding site between Runx3 3′UTR and miR-194-5p. At present, the involvement of Runx3 in neuronal apoptosis induced by CCI has not been reported.

Silent information regulator 1 (Sirt1) is an NAD+-dependent protein deacetylase. As a multi-functional protein, it participates in the regulation of cell differentiation, ageing, and withering through the deacetylation of histones and non-histones, getting involved in cell death, oxidative stress and other important biological processes^31–36^. Sirt1 plays an essential role in endogenous neuroprotection, by deacetylating p53 and NFκB (p65), inhibiting apoptosis and inflammatory response, and reducing ischaemic brain damage^37^. By using the Ensemble database and applying the bioinformatics software JASPAR, a potential binding site between the Sirt1 promoter region and Runx3 was predicted.

We investigated the expression levels of circ_0000296, miR-194-5p, and Runx3 in neurons induced with CCI and study the possible modes of action between the above molecules and neuronal apoptosis induced by CCI. The research aims to reveal the new mechanism of neuronal apoptosis induced by CCI and also provide novel ideas for targeted molecular therapy of CCI.

## Results

### Effect of CCI to neuronal apoptosis and relative expression levels of circ_0000296 and miR-194-5p

**Fig. 1:**
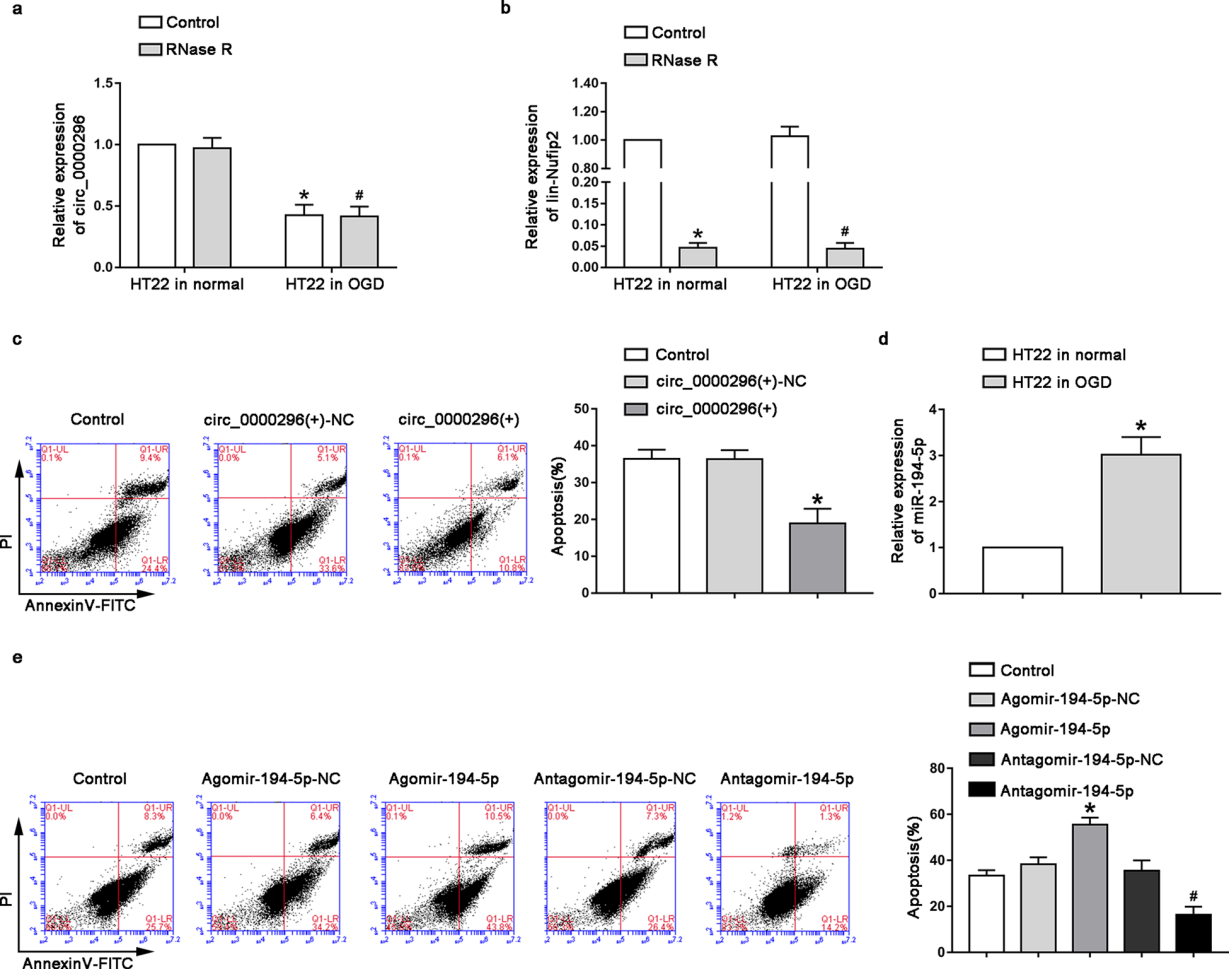
Effect of CCI to neuronal apoptosis and relative expression levels of circ_0000296 and miR-194-5p. **a** Relative expression of circ_0000296 in OGD-induced HT22 cells and HT22 cells in normal with RNase R treatment by quantitative real-time PCR. Data represented as mean ± SD. (*n* = 5), *P < 0.05 vs. normal HT22 cells with control group. #*P* < 0.05 vs. normal HT22 cells group with RNase R treatment. **b** Relative expression of lin-Nufip2 in OGD-induced HT22 cells and HT22 cells in normal with RNase R treatment. Data represented as mean ± SD (*n* = 5). *P < 0.05 or #*P* < 0.05 vs. control group. **c** Flow cytometry analysis of OGD-induced HT22 cells with overexpression of circ_0000296 with annexin V/PI staining. Data represented as mean±SD (*n* = 3). *P < 0.05 vs. circ_0000296(+)-NC group. **d** Relative expression of miR-194-5p in OGD-induced HT22 cells and HT22 cells in normal by quantitative real-time PCR. Data represented as mean±SD (*n* = 5). *P < 0.05 vs. OGD-induced HT22 cells control group. **e** Flow cytometry analysis of OGD-induced HT22 cells with transfection of miR-194-5p with annexin V/PI staining. Data represented as mean ± SD (*n* = 3). *P < 0.05 vs. Agomir-194-5p-NC group, #*P* < 0.05 vs. Antagomir-194-5p-NC group.

Compared with the normal HT22 cells, the expression level of circ_0000296 in the oxygen-glucose-deprived HT22 cells was significantly reduced (Fig. 1a, *P* < 0.05). RNase R, a restriction RNA exonuclease, degrades linear RNA but does not affect circular RNA. Both normal and oxygen-glucose-deprived HT22 cells were treated with RNase R to assess the expression level of circ_0000296. It was found that RNase R treatment did not alter the expression level of circ_000096 significantly in both cell types (Fig. 1a) compared with that in the control subgroup (without RNase R treatment), suggesting that circ_0000296 was resistant to RNase R treatment. However, the expression level of lin-Nufip2 (circ_0000296 parental gene) significantly reduced (Fig. 1b, *P* < 0.05) in both normal and oxygen-glucose-deprived HT22 cells upon RNase R treatment. In order to study the effect of circ_0000296 on neuronal apoptosis induced by CCI, HT22 cells overexpressing circ_0000296 were subjected to oxygen-glucose deprivation (OGD) for 48 h. Compared with the circ_0000296(+)-NC group, the apoptosis rate in the circ_0000296(+) group was significantly reduced (Fig. 1c, *P* < 0.05). The expression level of miR-194-5p in both normal and oxygen-glucose-deprived HT22 cells was evaluated, and the results showed that miR-194-5p expression in the oxygen-glucose-deprived group was significantly increased (Fig. 1d, *P* < 0.05). miR-194-5p-overexpressing and miR-194-5p-silenced HT22 cells were oxygen-glucose deprived for 48 h to further study the effect of miR-194-5p on neuronal apoptosis induced by CCI. The apoptosis rate of the Agomir-194-5p group increased significantly than in the Agomir-194-5p-NC group; compared with the Antagomir-194-5p-NC group, the apoptosis rate of the Antagomir-194-5p group decreased significantly (Fig. 1e, *P* < 0.05).

### circ_0000296 specifically bound to miR-194-5p to regulated neuronal apoptosis induced by CCI

**Fig. 2:**
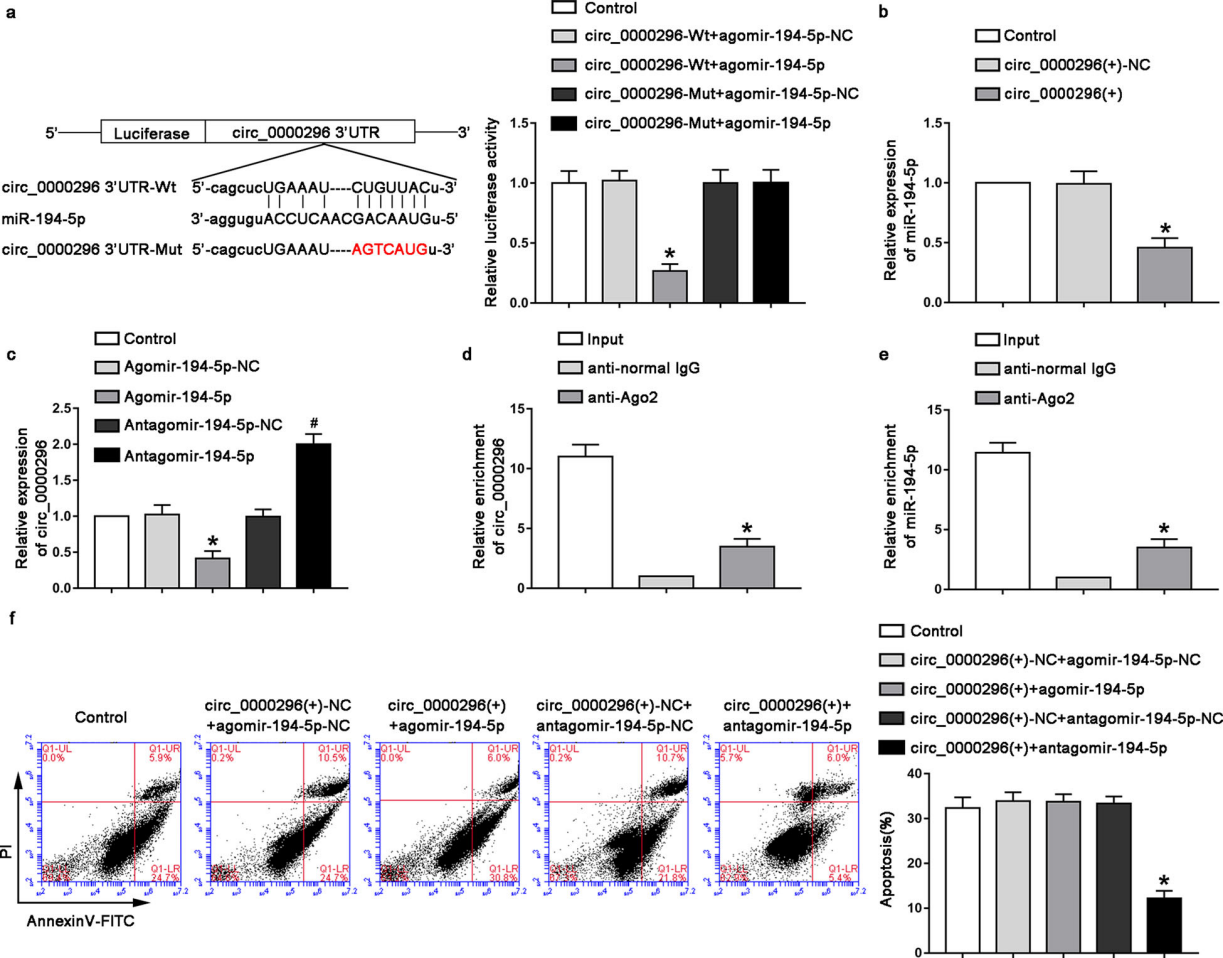
miR-194-5p targetd circ_0000296 and reversed the effects of circ_0000296 on apoptosis of neuron in vitro. **a** The predicted miR-194-5p-binding site in circ_0000296 (circ_0000296-Wt) and the designed mutant sequence (circ_0000296-Mut) were indicated. Dual-luciferase reporter assay of HT22 cells cotransfected with circ_0000296-Wt or circ_0000296-Mut and Agomir-194-5p or the Agomir-194-5p-NC. Data represented as mean ± SD. (*n* = 5), *P < 0.05 vs. circ_0000296-Wt+agomir-194-5p-NC group. **b** Relative expression levels of miR-194-5p were measured by qRT-PCR after circ_0000296 overexpression Data represented as mean ± SD. (*n* = 5), *P < 0.05 circ_0000296(+)-NC group **c** Relative expression levels of circ_0000296 were measured by qRT-PCR after miR-194-5p overexpression or knockdown. Data represented as mean ± SD. (*n* = 5), *P < 0.05 vs. Agomir-194-5p-NC group. #P < 0.05 vs. Antagomir-194-5p-NC group. **d**, **e** miR-194-5p was identified in circ_0000296-RISC complex. Relative expression levels of circ_000029 and miR-194-5p were measured by qRT-PCR. Data represented as mean ± SD. (*n* = 5, each group). *P < 0.05, vs. anti-normal IgG group. **f** Flow cytometry analysis of OGD-induced HT22 cells cotransfected with circ_0000296 and miR-194-5p with annexin V/PI staining. Data represented as mean ± SD. (*n* = 3, each group) *P < 0.05 vs. circ_0000296(+)-NC + antagomir-194-5p-NC.

We predicted a possible binding site between circ_000296 and miR-194-5p by a bioinformatics database (starBase). The dual-luciferase reporter assay determined if there was a targeted binding effect between circ_000296 and miR-194-5p. It was found that the luciferase activity in the circ_0000296-Wt+agomir-194-5p group was significantly reduced compared with that in the circ_0000296-Wt+agomir-194-5p-NC group (Fig. 2a, *P* < 0.05). Following the overexpression of circ_0000296, the expression of miR-194-5p increased significantly (Fig. 2b, *P* < 0.05); consequently, after the overexpression of miR-194-5p, the expression of circ_0000296 decreased significantly (Fig. 2c, *P* < 0.05). Further detection using RNA binding protein immunoprecipitation (RIP) experiments revealed that in the anti-Ago2 group, circ_0000296 and miR-194-5p were upregulated (Fig. 2d, e, *P* < 0.05), indicating that circ_0000296 and miR-194-5p existed simultaneously in RNA-induced silencing complex (RISC). To further investigate whether circ_0000296 regulated neuronal apoptosis induced by CCI via regulating miR-194-5p, we performed rescue experiments. The results showed that compared with that in the circ_0000296(+)-NC + antagomir-194-5p-NC group, the apoptosis rate in the circ_0000296(+)-NC + antagomir-194-5p group was significantly reduced (Fig. 2f, *P* < 0.05). There was no significant change in the apoptosis rate of the circ_0000296(+)+agomir-194-5p group compared with that of the circ_0000296(+)-NC + agomir-194-5p-NC group.

### Runx3 was the target gene of miR-194-5p which inhibited neuronal apoptosis induced by CCI

**Fig. 3:**
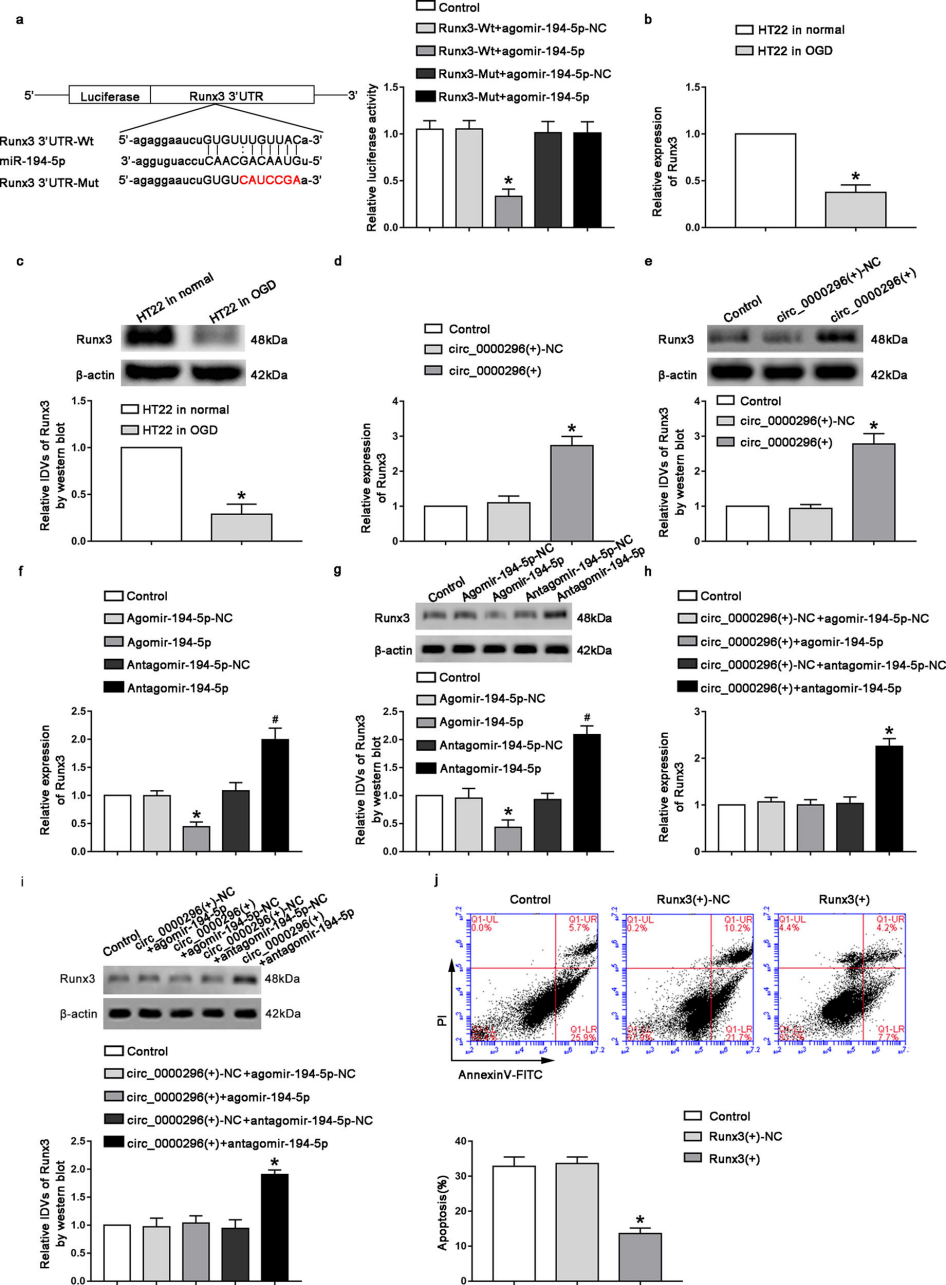
Runx3 was involved in circ_0000296 and miR-194-5p-mediated neuronal apoptosis by (CCI. **a** The predicted miR-194-5p-binding site in Runx3 (Runx3-Wt) and the designed mutant sequence (Runx3-Mut) were indicated. Dual-luciferase reporter assay of HT22 cells cotransfected with Runx3-Wt or Runx3-Mut and Agomir-194-5p or the Agomir-194-5p-NC. Data represented as mean ± SD. (*n* = 3), *P < 0.05 vs. Runx3-Wt+agomir-194-5p-NC group. **b**, **c** Relative expression levels of Runx3 were measured by qRT PCR and western blot. Data represented as mean ± SD. (*n* = 3), *P < 0.05 vs. HT22 in normal group. **d**, **e** Relative expression levels of Runx3 were measured by qRT-PCR and western blot after circ_0000296 overexpression. Data represented as mean ± SD. (*n* = 3), *P < 0.05 circ_0000296(+)-NC group. **f**, **g** Relative expression levels of Runx3 were measured by qRT-PCR and western blot after miR-194-5p overexpression or knockdown. Data represented as mean ± SD. (*n* = 3), *P < 0.05 vs. Agomir-194-5p-NC group. #*P* < 0.05 vs. Antagomir-194-5p-NC group. **h**, **i** Relative expression levels of Runx3 were measured by qRT-PCR and western blot after co-transfection with circ_0000296 and miR-194-5p. Data represented as mean ± SD. (*n* = 3, each group) *P < 0.05 vs. circ_0000296(+)-NC + antagomir-194-5p-NC group. **j** Flow cytometry analysis of OGD-induced HT22 cells after Runx3 overexpression with annexin V/PI staining. Data represented as mean ± SD. (*n* = 3, each group) *P < 0.05 vs. Runx3(+)-NC group.

Using bioinformatics database starBase, we predicted a possible miR-194-5p-binding site in Runx3. In order to confirm whether there is a targeted binding effect between Runx3 and miR-194-5p, we performed dual-luciferase reporter gene experiments. After cotransfection of Runx3-Wt and agomir-194-5p, the luciferase activity was significantly reduced (Fig. 3a, *P* < 0.05). Also, the endogenous expression level of Runx3 in the oxygen deprivation group was significantly reduced (Fig. 3b, c, *P* < 0.05) compared with that in the normal HT22 cells. We further investigated whether Runx3 is involved in the regulation of circ_0000296 and miR-194-5p in neuronal apoptosis induced by CCI. After the overexpression of circ_0000296, the mRNA and protein expression of Runx3 in the circ_0000296(+) group increased significantly compared with that in the circ_0000296(+)-NC group (Fig. 3d, e, *P* < 0.05). After transfection with miR-194-5p, compared with those of the Agomir-194-5p-NC group, mRNA and protein expression levels of Runx3 in the Agomir-194-5p group were significantly reduced (Fig. 3f, g, *P* < 0.05). Furthermore, the mRNA and protein expression levels of Runx3 in the Antagomir-194-5p group were significantly increased compared with those in the Antagomir-194-5p-NC group (Fig. 3f, g, P < 0.05). After the co-transfection of circ_0000296 and miR-194-5p, mRNA and protein expression of Runx3 in the circ_0000296(+)+antagomir-194-5p group increased significantly compared with that in the circ_0000296(+)-NC + antagomir-194-5p-NC group (Fig. 3h, i, *P* < 0.05). Moreover, we examined the effect of Runx3 overexpression on neuronal apoptosis induced by CCI. Compared with that of the Runx3(+)-NC group, the apoptosis rate of the Runx3(+) group decreased significantly (Fig. 3j, *P* < 0.05).

### Sirt1 and Runx3 inhibited neuronal apoptosis induced by CCI

**Fig. 4:**
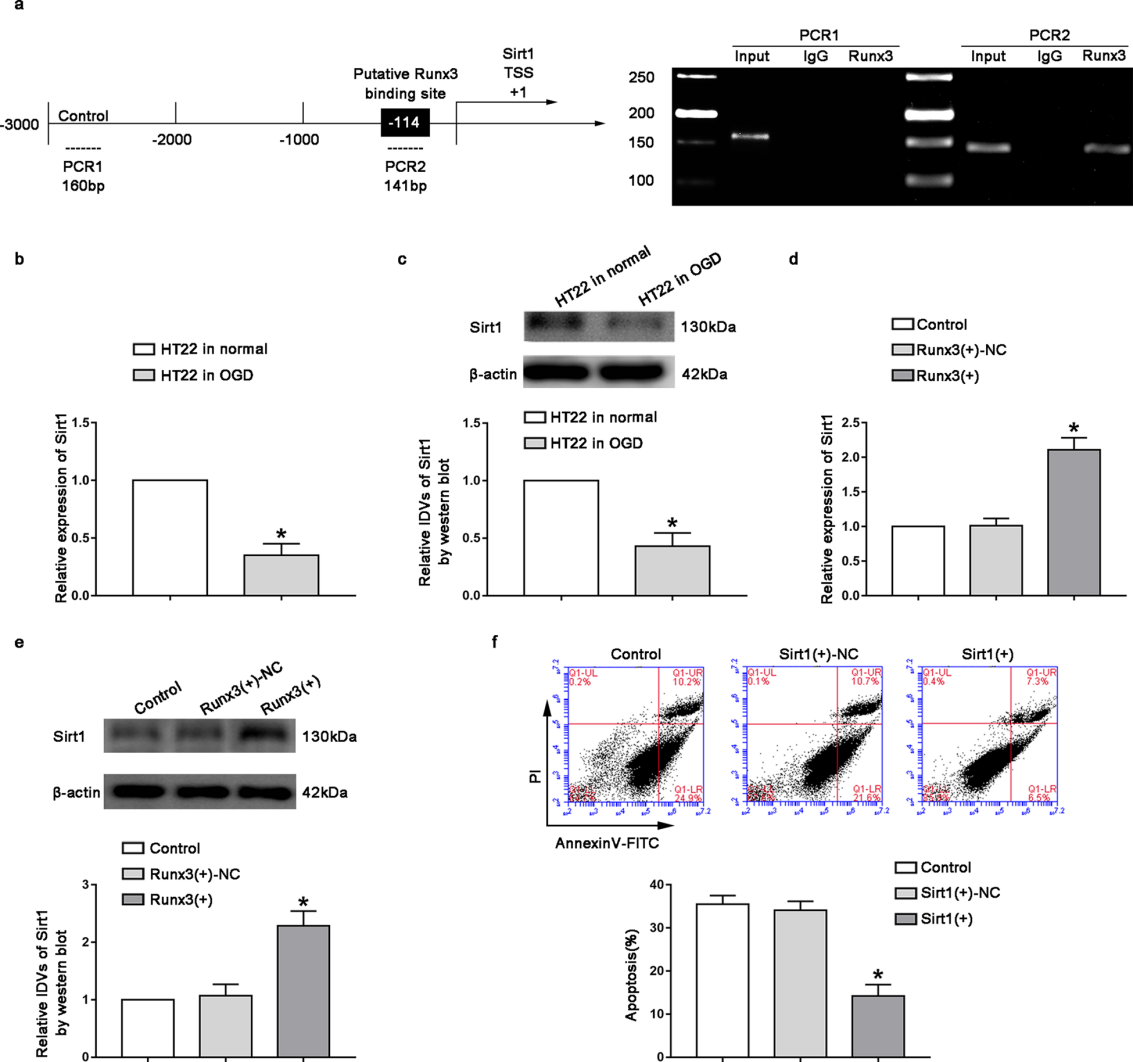
Runx3 bound to the promoter regions of Sirt1, Sirt1 inhibited neuronal apoptosis by CCI. **a** Runx3 bound to the promoter of Sirt1 in HT22 cell lines. Immunoprecipitated DNA was amplified by PCR. **b**, **c** Relative expression levels of Sirt1 were measured by qRT-PCR and western blot. Data represented as mean ± SD. (*n* = 3), * *P* < 0.05 vs. HT22 in normal group. **d**, **e** Relative expression levels of Sirt1 were measured by qRT-PCR and western blot after Runx3 overexpression Data represented as mean ± SD. (*n* = 3), *P < 0.05 vs. Runx3(+)-NC group. **f** Flow cytometry analysis of OGD-induced HT22 cells after Sirt1 overexpression with annexin V/PI staining. Data represented as mean ± SD. (*n* = 3, each group) *P < 0.05 vs. Sirt1(+)-NC group.

JASPAR showed that there was a potential Runx3 binding site in the promoter region of Sirt1, which was located 114 bp upstream of the Sirt1 transcription initiation site. We designed primers upstream and downstream of the predicted binding site, also designing primers for at least 1000 bp upstream of the binding site as a negative control. A ChIP experiment was conducted to verify whether Runx3 directly bound to the promoter region of Sirt1. The results showed Runx3 binding to Sirt1 at the predicted binding site, but no binding was observed at the control region (Fig. 4a). The endogenous expression level of Sirt1 in the oxygen-sugar deprivation group was significantly reduced compared with that in the normal HT22 cells (Fig. 4b, c, *P* < 0.05). However, after Runx3 overexpression, Sirt1 mRNA and protein expression levels in the Runx3(+) group were significantly increased compared to those in the Runx3(+)-NC group (Fig. 4d, e, *P* < 0.05). When we further explored the effect of Sirt1 overexpression on neuronal apoptosis induced by CCI, we found that apoptosis rate in the Sirt1(+) group decreased significantly compared with that in the Sirt1(+)-NC group (Fig. 4f, *P* < 0.05).

### miR-194-5p targeted Runx3 3′-UTR to regulated Sirt1 expression and CCI-induced neuronal apoptosis

**Fig. 5:**
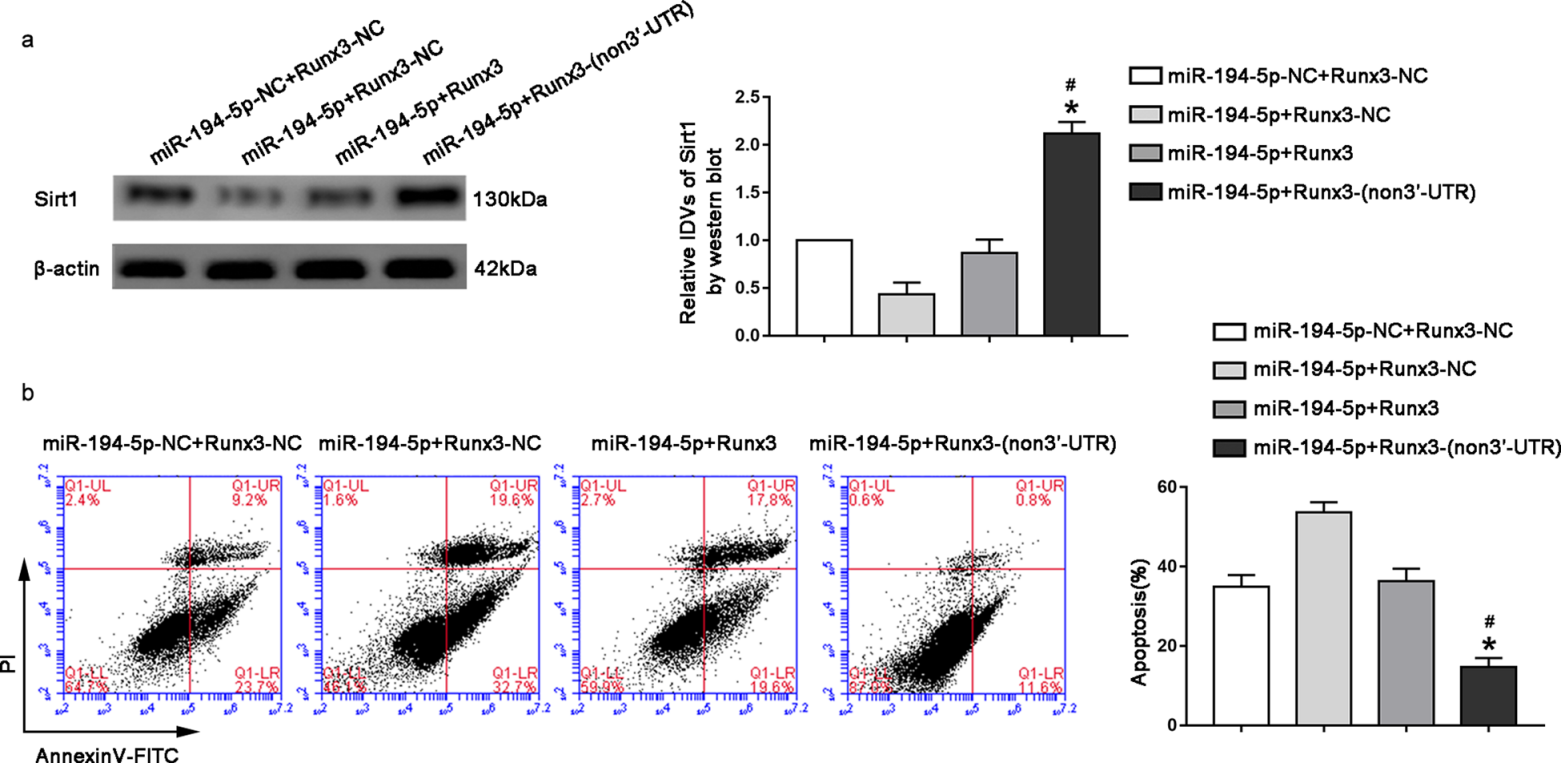
Overexpression of miR-194-5p impaired Runx3-induced attenuation of CCI-induced neuronal apoptosis by targeting 3′-UTR of Runx3. **a** Sirt1 protein levels in CCI-induced HT22 cells cotransfected with miR-194-5p and Runx3/Runx3-(non-3′UTR). Data represented as mean ± SD. (*n* = 3, each group) *P < 0.05 vs. miR-194-5p + Runx3-NC group, #*P* < 0.05 vs. miR-194-5p + Runx3 group. **b** Flow cytometry analysis of CCI-induced HT22 cells cotransfected with miR-194-5p and Runx3/Runx3-(non-3′UTR) with annexin V/PI staining. Data represented as mean ± SD. (*n* = 3, each group) *P < 0.05 vs. miR-194-5p + Runx3-NC group, #*P* < 0.05 vs. miR-194-5p + Runx3 group.

This study further investigated whether miR-194-5p targets the 3′UTR end of Runx3 to regulate neuronal apoptosis and Sirt1 expression levels induced by CCI. Runx3 and miR-194-5p transfection in HT22 cells induced with CCI showed that the expression level of Sirt1 in the miR-194-5p + Runx3-(non3′-UTR) group increased significantly compared with that in both miR-194-5p + Runx3-NC and miR-194-5p + Runx3 groups (Fig. 5a, *P* < 0.05). When we further explored the effect of co-transfection of miR-194-5p and Runx3 on neuronal apoptosis induced by CCI, we found that apoptosis rate in the miR-194-5p + Runx3-(non3′-UTR) group decreased significantly compared with that in the miR-194-5p + Runx3 group (Fig. 5b, *P* < 0.05), but compared with the miR-194-5p + Runx3-NC group, the apoptosis level in the miR-194-5p + Runx3-(non3′UTR) group decreased more drastically (Fig. 5b, *P* < 0.05).

### Overexpression of circ_0000296 antagonised neuronal apoptosis in a CCI mouse model

**Fig. 6:**
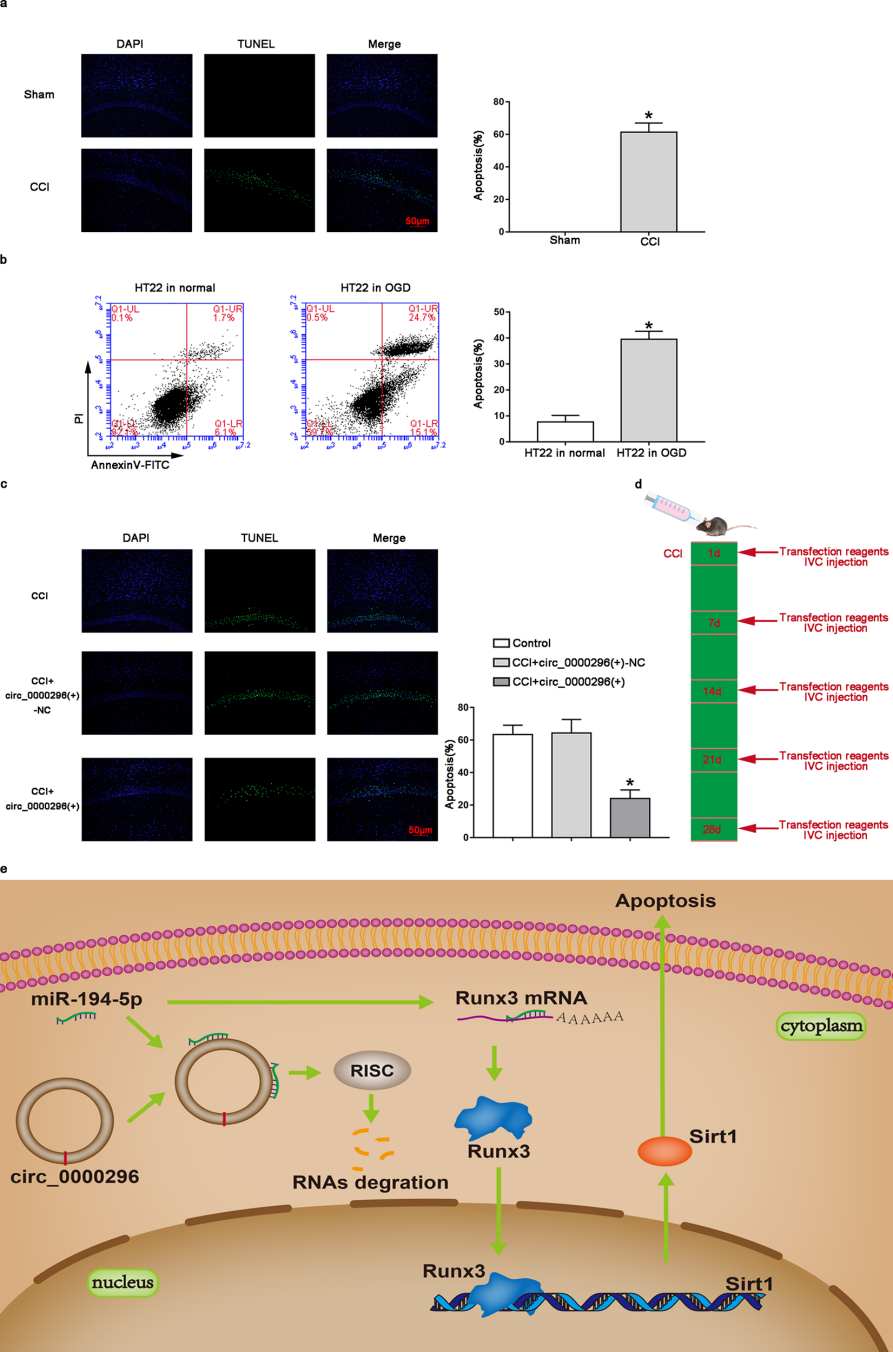
Overexpression of circ_0000296 against CCI induced neuronal apoptosis in vivo. **a** Terminal deoxynucleotidyl transferase (TdT)-mediated dUTP-biotin nick end labelling (TUNEL) assay (×200, scale bar = 50 μm) sections of the hippocampus CA1 region of Sham (*n* = 15) and CCI (*n* = 20) group. Data represented as mean ± SD.*P < 0.05 vs. Sham group. **b** Flow cytometry analysis of CCI-induced HT22 cells with annexin V/PI staining. Data represented as mean ± SD. (*n* = 3, each group). *P < 0.05 vs. HT22 in normal group. **c** TUNEL assay (×200, scale bar = 50 μm) sections of the hippocampus CA1 region of CCI (*n* = 20), CCI + circ_0000296(+)-NC (*n* = 20) and CCI + circ_0000296(+) group (*n* = 15). Data represented as mean ± SD. *P < 0.05 vs. CCI + circ_0000296(+)-NC group. **d** Experimental schedule to explore the effects of circ_0000296 on CCI-induced neuronal damage in vivo. **e** The schematic cartoon of the mechanism of circ_0000296/miR-194-5p/Runx3/Sirt1 axis regulating neuronal apoptosis.

TUNEL experiments were performed on the hippocampal CA1 tissue in the sham operation group and CCI group. The results of TUNEL staining-positive cells (i.e., apoptotic cells) were green, and the apoptosis rate in the CCI group was significantly increased (*P* < 0.05; apoptosis rate = number of TUNEL staining-positive neurons in the CA1 area/total number of neurons in the CA1 area) (Fig. 6a). Compared with that in the normal HT22 cells, the level of apoptosis in the OGD HT22 cell group was significantly increased (Fig. 6b, *P* < 0.05). In order to study whether overexpression of circ_0000296 can antagonize neuronal apoptosis in a CCI mouse model, we injected the circ_0000296(+) with vivo transfection reagent into the lateral ventricle of the mouse model of CCI. The results showed that compared with the CCI + circ_0000296(+)-NC group, the apoptosis rate of the CCI + circ_0000296(+) group was significantly reduced (Fig. 6c, *P* < 0.05). The schedule of circ_0000296 in vivo transfection on neuronal damage induced by CCI is shown in Fig. 6d. A schematic representation of the mechanism of mmu_circ_0000296 activity through the miR-194-5p/Runx3/Sirt1 pathway to regulate neuronal apoptosis induced by CCI is shown in Fig. 6e.

## Discussion

This study revealed that in HT22 cells and hippocampus induced with CCI, circ_0000296, Runx3, Sirt1 expressions were low, and miR-194-5p expression was high. After the overexpression of circ_0000296, overexpression of Runx3, overexpression of Sirt1, and silence of miR-194-5p, significantly inhibited neuronal apoptosis induced by CCI. circ_0000296 bound to miR-194-5p at a specific sequence; miR-194-5p bound to the 3′UTR region of Runx3 mRNA through the same sequence; Runx3 directly bound to the promoter region of Sirt1, enhancing its transcriptional activity. This demonstrated that mmu_circ_0000296 played an important role in regulating neuronal apoptosis induced by CCI through the miR-194-5p/Runx3/Sirt1 pathway.

Studies have confirmed that circRNAs are involved in regulating the development of ischaemic cerebrovascular disease^38–40^. circDLGAP4 binds to miR-143 and inhibits its activity, regulates the expression of HECTD1 and thus affects the dedifferentiation of endothelial cells into mesenchymal cells, reduces cerebral infarct area and blood-brain barrier damage^41^. Previous studies have shown that circular RNA is involved in regulating apoptosis in various types of cells, including neurons. In the development of Alzheimer’s disease, circ_0000950 negatively regulates the neuronal apoptosis of miR-103^42^. Overexpression of circVMA21 reduces inflammatory cytokine-induced nucleus pulposus cells (NPCs) through the miR-200c-XIAP pathway^43^. Hsa_circ_0010729 regulates the apoptosis of vascular endothelial cells by targeting the miR-186/HIF-1α axis^44^. Here, we found that in HT22 cells and hippocampal tissues treated with CCI, circ_0000296 was underexpressed, and the overexpression of circ_0000296 significantly inhibited CCI-induced neuronal apoptosis.

Studies have confirmed that miR-194-5p participates in the origin and development of various diseases, such as tumours, ischaemic injuries, and neurodegenerative diseases. In ovarian cancer, knocking out miR-194-5p promotes the expression of IGF1R and PPFIBP1, which in turn promotes the proliferation, migration, and invasion of ovarian cancer cells^45^. miR-194-5p promotes the survival and proliferation of renal tubular epithelial cells induced by ischaemia-reperfusion through the targeted inhibition of Rheb expression, reduces inflammation and oxidative stress, and exerts renal protection^46^. In addition, miR-194-5p also participates in the regulation of apoptosis in certain diseases. In Alzheimer’s disease, miR-194 targets Nrn1 and reduces the PI3K/Akt signalling pathway activity, promoting hippocampal neuronal apoptosis^47^. In bladder cancer, miR-194-5p targeting in combination with CCND2 significantly inhibits bladder tumour cell proliferation and promotes bladder tumour cell apoptosis^48^. In acute myeloid leukaemia (AML), miR-194-5p targets DNMT3A, inhibits the proliferation, migration, and invasion of AML cells, and promotes apoptosis^49^. Here, we demonstrated that miR-194-5p was highly expressed in HT22 cells and hippocampus induced with CCI and silencing miR-194-5p expression inhibited CCI-induced neuronal apoptosis significantly.

Since the overexpression of circ_0000296 and miR-94-5p silencing significantly inhibited neuronal apoptosis induced by CCI, this study further explored whether there was a direct binding between circ_0000296 and miR-194-5p. Studies have shown that circRNAs can combine with miRNAs to regulate the expression of their target genes, which in turn affects the pathological process of ischaemic hypoxic diseases. Circ_002664 directly targets miR-182-5p, thereby regulating endoplasmic reticulum stress and neuronal apoptosis after cerebral ischaemia and reperfusion in patients with acute stroke^50^. Has_circ_0072309 reduces neuronal apoptosis in ischaemic stroke by targeting the miR-100/mTOR axis^51^. circPKT2 promotes neuronal apoptosis by negatively regulating miR-29b and regulating SOCS-1-JAK2/STAT3-IL-1β signalling pathway^52^. Hsa_circ_0007623, when combined with miR-297, promotes cardiac repair after acute myocardial ischaemia and improves cardiac function^53^. The present study revealed that circ_0000296 and miR-194-5p had potential binding sites, by using the starBase database, and dual-luciferase reporter gene experiments confirmed that circ_0000296 and miR-194-5p had a targeted binding effect. RIP experiments confirmed that circ_0000296 and miR-194-5p formed a RISC complex. Also, the overexpression of circ_0000296 significantly downregulated the expression of miR-194-5p. Overexpression of circ_0000296 simultaneously silenced miR-194-5p, which significantly reduced CCI-induced neuronal apoptosis. Double overexpression of circ_0000296 and miR-194-5p reversed the decrease in the rate of CCI-induced neuronal apoptosis caused by overexpression of circ_0000296 simultaneously silenced miR-194-5p, indicating that circ_0000296 regulates CCI-induced neuronal apoptosis by regulating miR-194-5p.

This study further demonstrated that Runx3 was underexpressed in HT22 cells and hippocampus induced with CCI, while its overexpression significantly inhibited neuronal apoptosis induced by CCI. Runx3 belongs to the family of Runt-related genes. It not only regulates the development of the central nervous system and participates in the formation of proprioceptive circuits^54^, but also acts as a tumour suppressor to regulate the occurrence and development of tumours^55–57^. Another study confirmed that hypoxia reduces the expression and silences the tumour-suppressive function of Runx3^58,59^. Runx3 can also conduct signal transduction through β2-nAChR to promote the decrease of the number and activity of NK (natural killer) cells in the brain of patients with cerebral ischaemia^60^. One of the functions of miRNAs is to target the 3′UTR end of mRNAs through complementary pairing, inhibit their translation, and regulate the expression of their target genes at the post-transcriptional level. Existing research have confirmed that miR-194-5p binds to target genes and plays a variety of biological functions. For example, miR-194-5p regulates FOXA1 by targeting and reversing the apoptosis and cell cycle arrest of hepatocellular carcinoma induced by MCM3AP-AS1 silencing^61^. miR-194-5p binds to FLI1 and downregulates its expression to increase the permeability of the blood tumour barrier^62^. In the current study, based on the application of biological information software starBase to predict the binding site of Runx3 and miR-194-5p, the dual-luciferase reporter gene experiment confirmed Runx3 as the target gene of miR-194-5p. Further experiments proved that miR-194-5p silencing inhibited neuronal apoptosis induced by CCI by upregulating the expression of Runx3 at mRNA and protein levels.

The results of this study showed that in HT22 cells induced with CCI, overexpression of circ_0000296 significantly increased the expression of Runx3. Overexpression and silencing of miR-194-5p significantly reduced or increased the expression of Run3, respectively. Further experiments revealed that circ_0000296 overexpression, while miR-194-5p knockdown, significantly increased Runx3 expression, while double co-overexpression of circ_0000296 and miR-194-5p, did not alter Run3 expression significantly. The above results suggested that the overexpression of circ_0000296 occurred via the downregulation of miR-194-5p and the subsequent upregulation of Runx3 expression, which played a role in inhibiting neuronal apoptosis induced by CCI.

Sirt1 is a nicotinamide-adenine dinucleotide-dependent histone deacetylase that delays ageing and plays a role in cellular oxidative stress injury (OSI)^63,64^. Studies have shown that Sirt1 can directly or indirectly participate in the occurrence and development of ischaemic hypoxic diseases^65,66^ and as a neuroprotective factor, reduce ischaemic brain damage^67^. Sirt1 protects against ischaemia-reperfusion-induced cardiomyocyte apoptosis through the ERK1/2/Homer1a pathway^68^. Sirt1 inhibits oxidative stress and apoptosis in HT22 cells through the FOXO1/PGC-1α signalling pathway and reduces oxygen and glucose deprivation/reperfusion-induced neuronal damage^69^. Oestrogen can protect neurons from death through the SIRT1/AMPK pathway, thereby preventing ischaemic stroke^70^. The results of this study showed that Sirt1 was underexpressed in HT22 cells and hippocampal tissues induced with CCI. Overexpression of Sirt1 significantly inhibited neuronal apoptosis induced by CCI. Studies have shown that Runx3 regulates the expression of various target genes and various biological functions. For example, it targets the promoter region of KDM2A, increasing its expression consequently inhibiting the proliferation and migration of gastric cancer cells^71^. Runx3 inhibits the transcription of ZO-1,occludin, and claudin-5 by binding to specific sites on ZO-1,occludin, and claudin-5 promoters, thereby enhancing the permeability of the blood tumour barrier^72^. In the present study, we used the Ensemble database and the bioinformatics software JASPAR to predict the potential binding sites of Runx3 and Sirt1 promoter region, and ChIP experiments confirmed that Runx3 could directly bind to Sirt1 promoter region, improving promoter region activity and Sirt1 transcription. It was further found that Runx3 significantly inhibited CCI-induced neuronal apoptosis by targeting Sirt1 and upregulating Sirt1 expression. In addition, rescue experimental results revealed that, overexpression of miR-194-5p reversed Runx3-induced upregulation of Sirt1 expression and the anti-apoptotic effect of Sirt1. The above results confirmed that miR-194-5p negatively regulated Runx3 expression and participated in the transcriptional regulation of Sirt1 via Runx3. Finally, we performed in vivo transfection experiments. circ_0000296 overexpression plasmid was injected into CCI mice through the lateral ventricle. The expression of miR-194-5p was significantly decreased, and the expression of circ_0000296, Runx3, and Sirt1 was significantly increased. Apoptosis rate of hippocampal neurons in mice with CCI decreased significantly.

In summary, this study demonstrated for the first time that in HT22 cells induced with CCI, circ_0000296 and Runx3 were underexpressed and miR-194-5p was highly expressed. Overexpression of circ_0000296 downregulated the expression of miR-194-5p and attenuated negative regulation of Runx3 via miR-194-5p, causing the upregulation of Runx3 which increased the transcription of Sirt1 and inhibited the apoptosis of hippocampal neurons induced by CCI. Therefore, circ_0000296 plays an important regulatory role in the hippocampal neuronal apoptosis induced by CCI through the miR-194-5p/Runx3/Sirt1 axis. The results of this study might provide new ideas and strategies for the study of pathogenesis and treatment of CCI.

## Materials and methods

### Cell culture and OGD

HT22 cell line was purchased from American Type Culture Collection. HT22 cells are routinely cultured in a humidified incubator with 5% CO_2_ at 37 °C. The medium is DMEM high glucose culture containing 10% foetal bovine serum. To study the effect of CCI on HT22 cells, we divide up the cells into two groups: control group and OGD group. We use glucose-free medium to cultivate the OGD group in a tri-gas incubator (SANYO, Osaka, Japan) at 37 °C with 3% O_2_, 5% CO_2_ for 48 h. The control group replaced the old medium with fresh DMEM medium, and cultured in a humidified incubator containing 5% CO_2_ and 95% air at 37 °C for 48 h.

### Animal model design

Adult male C57BL/6J mice aged 12–14 weeks were used to make CCI models. All experiments were approved by the Ethics Committee of China Medical University. ARRIVE guidelines were followed. The mice were individually housed in an environment with suitable humidity and temperature and were given standard diet. In order to identify the effect of CCI on the hippocampal neurons in mice, 24–29 grams of mice were randomly divided into two groups: sham operation group (*n* = 15) and CCI group (*n* = 20). In order to observe the effect of circ_0000296 on hippocampal neuron apoptosis, the mice weighing 24–29 g were randomly divided into three groups: CCI group (*n* = 20), CCI + circ_0000296(+)-NC group (*n* = 20), CCI + circ_0000296(+) group (*n* = 15).

### Mouse model of CCI and transfection in vivo

Using 1.5% isoflurane anesthetizes the mice. The mice were anesthetized with 1.5% isoflurane. The operation was started after confirming that the mouse was motionless and had no pinch reflex. The skin of the mouse was cut along the midline of the cervical to expose the common carotid artery, which was wrapped sheaths. In the sham group, the bilateral common carotid arteries were exposed without implantation of ACs (Research Instruments SW, Escondido, USA); in the CCI group, ACs (Research Instruments SW, Escondido, USA) were placed at the bilateral common carotid arteries. Use a heating pad to maintain the rectal temperature. CCI-induced mice received intracerebraoventricular (IVC) injections of circ_0000296 plasmid via DNA reagent (Engreen Biosystem Co, Ltd, China). On the first day of CCI treatment and every 7 days after the operation, each mouse was injected with 1 μl plasmid intracerebroventricularly to maintain the transfection effect in vivo.

### Real-time quantitative polymerase chain reaction (qRT-PCR)

Trizol reagent (Life Technologies Corporation, Carlsbad, CA, USA) was used to extract total RNA from the CA1 area of the hippocampus and HT22 cells. A Nanodrop spectrophotometer (ND-100, Thermo Fisher Scientific, Waltham, MA, USA) was used to measure RNA concentration and quality at a ratio of 260/280 nm. RNase R was used to confirm the existence of circ_0000296, and eliminated the influence of liner RNAs. The primers of circ_0000296, Runx3, Sirt1 and β-actin were synthesized from Shanghai ShineGene. One-Step SYBR Prime Script RT-PCR Kit (Takara Biomedical Technology, Dalian, China) were used for measurement of circ_0000296, Runx3 and Sirt1. β-actin was used as the endogenous control.

circ_0000296: Forward 5′-AAAGGAATGATAGCTGGGGTTC-3′,

Reverse 5′-GGTTGGTAGCTTCATCAGAACTG-3′;

Runx3: Forward 5′-GACCGCTTTGGAGACCTGC-3′,

Reverse 5′-TCTGAGGAGCCTTGGATTGG-3′;

Sirt1: Forward 5′-AAAGTGATGACGATGACAGAACG-3′,

Reverse 5′-GCCAATCATGAGATGTTGCT-3′;

β-actin: Forward 5′-GAGACCTTCAACACCCCAGC-3′,

Reverse 5′-ATGTCACGCACGATTTCCC-3′.

TaqMan MicroRNA Reverse Transcription kit and TaqMan Universal Master Mix II (Applied Biosystems, Foster City, CA, USA) were used to detect the expression of miR-194-5p and U6. The relative quantitative (2^−ΔΔCt^) method was applied to calculate the gene expression level. The primers are follows.

### Apoptosis analysis

The Annexin V: FITC Apoptosis Detection Kit (Southern Biotech, Birmingham, AL, USA) was used to detect the apoptosis of HT22 cells. After washing with cold PBS twice, cells were stained with Annexin V: FITC. The apoptosis degree of the cells was analyzed by flow cytometry (BD Biosciences, Palo Alto, CA, USA). The meaning of each area cells is as follows: Q1:(AnnexinV-FITC)-/PI+, the vast majority of cells in this area are dead cells. Q2:(AnnexinV + FITC)+/PI+, the cells in this area are late-stage apoptotic cells. Q3:(AnnexinV-FITC)+/PI−, the cells in this area are early-stage apoptotic cells. Q4:(AnnexinV-FITC)-/PI−, the cells in this area are live cells. We consider the cells in the Q2 plus Q3 regions as apoptotic cells.

The One Step TUNEL Apoptosis Assay Kit (Beyotime, Jiangsu, China) was used to detect neuronal apoptosis in the hippocampal CA1 area.

### RNA binding protein immunoprecipitation (RIP)

Lyse HT22 cells with the protease inhibitor, RNase inhibitor and total RNA lysis buffer in the EZMagna RIP kit (Millipore, MA, USA). Whole-cell lysate was incubated with RIP immunoprecipitation buffer containing mouse anti-Argonaute2 (Ago2) conjugated magnetic beads (Millipore, MA, USA) and mouse anti-IgG conjugated magnetic beads (Millipore, MA, USA). Samples were incubated with Proteinase K buffer and then immunoprecipitated RNA was isolated. Use NanoDrop (Thermo Scientific, Waltham, MA, USA) and RNA quality measurement bioanalyzer (Agilen Santa Clara, CA, USA) to evaluate RNA concentration and quality. The presence of the binding targets were demonstrated by qRT-PCR using respective primers mentioned earlier.

### Western blot analysis (WB)

The total protein of HT22 cells was extracted with RIPA lysis buffer ((Beyotime Institute of Biotechnology, Jiangsu, China) containing protease inhibitors. The same amount of proteins (40 μg) were transferred to PVDF membrane by SDS-PAGE electrophoresis. The membrane was placed in tris-buffered saline (TBS) containing 0.1% Tween-20 and 5% skimmed milk powder at room temperature for 2 h. And then the membranes were incubated overnight at 4 °C with primary antibodies as follows: Runx3 (1:800, Santa Cruz Biotechnology), Sirt1 (1:1000, Proteintech, Chicago, IL), and β-actin (1:10,000, Proteintech, Chicago, IL). The next day, the membrane was incubated with the secondary antibody (goat anti-mouse, 1:10,000, Proteintech, Chicago, IL) for 2 h at room temperature. Finally, these protein blots were detected by the Enhanced Chemiluminescence Kit (Santa Cruz Biotechnology) and ECL Detection Systems (Thermo Scientific, Beijing, China) and scanned using Chemi Imager 5500 V2.03. The integrated density values (IDVs) of the blots were calculated by Fluor Chen 2.0 software, with β-actin as an internal control.

### Chromatin immunoprecipitation (ChIP)

The Simple ChIP Enzymatic Chromatin IP Kit (Cell Signaling Technology, Danvers, MA, USA) was applied to ChIP assays based on the manufacturer’s instructions. Briefly, cells were first cross-linked with EBM-2 containing 1% formaldehyde for 10 min and then collected in a lysis buffer containing 1% phenylmethanesulfonyl fluoride (PMSF). In addition, the use of micrococcus nuclease digested chromatin. Immunoprecipitation was incubated with 3 μg of anti-Runx3 antibody (1:800, Santa Cruz Biotechnology). Protein G Agarose Beads were used for immunoprecipitation in each sample and incubated at 4 °C overnight with gently shaking. Then the DNA crosslink samples were reversed by 5 mol/l NaCl and Proteinase K at 65 °C for 2 h and then purified DNA.

Immunoprecipitated DNA was amplified by PCR using their specific primers as follows:

Control PCR1: forward 5′-AGGCGTGTCAGCTAAAAGGG-3′;

Reverse 5′-TGGATGTCAGTGGGTTGTGG-3′;

Sirt1 PCR2: Forward 5′-CCCGGCTTCCAAGGACATC-3′;

Reverse 5′-TTTCTCTGTGTAGCCCTGGC-3′.

### Vector construction and dual-luciferase experiment

The potential binding sequence and the corresponding mutant sequence of miR-194-5p in circ_0000296 and Runx3 3′-UTR were amplified by PCR and then cloned into the pmirGLO Dual-Luciferase miRNA Target Expression Vector (Promega, Madison, WI, USA) to construct wild type and mutation type luciferase reporter vectors (GenePharma, Shanghai, China). HT22 cells were cultured in 96-well plates and the cells were co-transfected with the above wild type and mutation type luciferase reporter vectors and agomir-194-5p or agomir194-5p-NC plasmid when they reached 50–70% confluence. After 48 h of transfection, luciferase activities were detected by Dual-Luciferase assay kit (Promega, Madison, WI, USA).

### Cell transfection

PcDNA3.1-circ_0000296 full-length plasmid (circ_0000296(+)) and non-targeting sequence (NC) (GenePharma, Shanghai, China), agomir-194-5p and antagomir-194-5p, and their respective nontargeting sequence (agomir-194-5p-NC or antagomir-194-5p-NC) (GenePharma, Shanghai, China) were synthesized. Runx3 (including 3′UTR) (Runx3(+)), Runx3 (without 3′UTR) (Runx3-(non-3′UTR)) plasmid, and non-targeting sequence (negative control, NC) (Runx3(+)-NC) (GenePharma, Shanghai, China); Sirt1 full length (Sirt1(+)) plasmid and corresponding non-targeting sequence (negative control, NC) (Sirt1(+)-NC) (GenePharma, Shanghai, China) were synthesized. HT22 cells were cultured into 24-well plates and transfected with the plasmids by Opti-MEM and Lipofectamine 3000 reagent when they reached 50–70%. G418 reagent was used continuously for 3–4 weeks to screen out G418-resistant HT22 cell clones. The transfection efficiency was analyzed using qRT-PCR and western blot. In order to determine the role of circ_0000296 in the apoptosis process of hippocampal neurons induced by CCI, the cells were divided into three groups: control group, circ_0000296(+)-NC group (transfected with pcDNA-NC plasmid group), circ_0000296(+) Group (transfected with circ_0000296 full-length plasmid). To determine the role of miR-194-5p in CCI-induced apoptosis of hippocampal neurons, the cells were divided into five groups: control group, agomir-194-5p-NC group, agomir-194-5p group, and antagomir- 194-5p-NC group, antagomir-194-5p group. To study the role of Runx3 in the apoptosis of hippocampal neurons induced by CCI. The cells were divided into three groups: control group, Runx3(+)-NC group, Runx3(+) group. In addition, in order to verify the basic mechanism by which circ_0000296 reduces miR-194-5p and induces apoptosis of hippocampal neurons, the cells were divided into five groups: control group, circ_0000296(+)-NC + agomir-194-5p-NC group, circ_0000296(+)+agomir-194-5p group, circ_0000296(+)-NC + antagomir-194-5p-NC group, circ_0000296(+)+antagomir-194-5p- group.

### Statistical methods

Data are expressed as mean ± SD. All statistical analyses were carried out by GraphPad Prism v5.01 (GraphPad, La Jolla, CA) with the Student’s *t* test or one-way ANOVA. Differences were considered to be statistically significant when *P* < 0.05.

## Supplementary information

Supplementary data

## Data Availability

The datasets used and/or analyzed during the current study are available from the corresponding author on reasonable request.
