# Supplementary figures and images for: Effect of circular RNA, mmu_circ_0000296, on neuronal apoptosis in chronic cerebral ischaemia via the miR-194-5p/Runx3/Sirt1 axis
Source: Cell Death Discov. 2021 May 29;7:124. doi: 10.1038/s41420-021-00507-y (PMC8164632; doi:10.1038/s41420-021-00507-y)

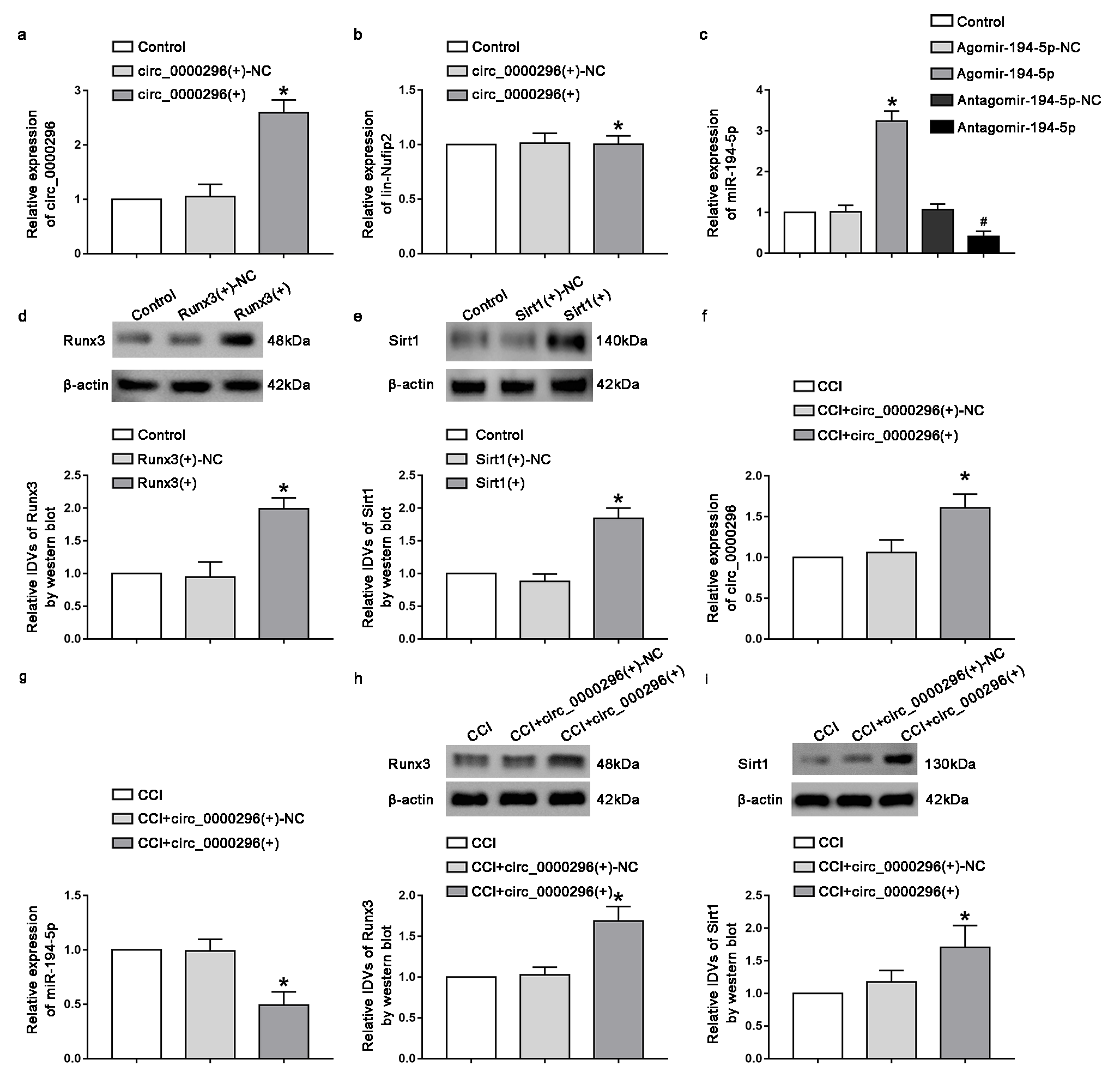

Supplement: Supplementary file 1 — Supplementary data [file 41420_2021_507_MOESM1_ESM.tif]
